# Heterogeneity in mental health change during the COVID‐19 pandemic in Germany: The role of social factors

**DOI:** 10.1002/smi.3181

**Published:** 2022-07-11

**Authors:** Dorota Reis, Kai Krautter, Alexander Hart, Malte Friese

**Affiliations:** ^1^ Saarland University Saarbrücken Germany

**Keywords:** COVID‐19, individual trajectories, latent change analysis, latent class growth analysis, life satisfaction, mental health, social factors

## Abstract

The COVID‐19 pandemic constitutes a prolonged global crisis, but its effects on mental health seem inconsistent. This inconsistency highlights the importance of considering the differential impact of the pandemic on individuals. There is some evidence that mental health trajectories are heterogeneous and that both sociodemographic and personal characteristics are associated with higher risk for mental health issues. By contrast, information on the role of social factors as potential determinants of initial reactions to the pandemic and on heterogeneous trajectories over time is lacking. We analysed seven assessments of a large‐scale (*N* = 2203) longitudinal study across 1.5 years, beginning in March 2020. Using self‐report data on mental health and life satisfaction, we applied latent change models to examine initial reactions and mean changes across the pandemic. In addition, we applied latent class growth analyses to investigate whether there were distinct groups with different patterns of change. Results showed that on average, levels of life satisfaction and anxiety decreased (*d* = −0.31 and *d* = −0.11, respectively), levels of depressive symptoms increased (*d* = 0.13), and stress levels remained unchanged (*d* = −0.01) during the first year of the pandemic. For each outcome, we identified four distinct mental health trajectories. Between 5% (for anxiety) and 11% (for life satisfaction) of the sample reported consistently high—and even increasing—impairments in mental health and well‐being. The trajectories of a sizeable number of people covaried with the course of the pandemic, such that people experienced better mental health when the number of COVID cases was low and when fewer restrictions were placed on public life. Low emotional support, high instrumental support, and the tendency to compare oneself with others were associated with more mental health issues. Findings show that whereas a substantial portion of people were largely unaffected by the pandemic, some individuals experienced consistently high levels of psychological distress. Social factors appear to play a crucial role in the maintenance of well‐being.

## 1 INTRODUCTION

The COVID‐19 outbreak constitutes a massive global crisis. In addition to afflicting people's physical integrity, the pandemic has placed a considerable psychological burden on the general public and has sparked pronounced interest in how the pandemic has affected mental health. Systematic reviews have revealed pooled prevalence estimates of about 30% for stress (Salari et al., 2020), 16%–34% for depression, and 15%–32% for anxiety (Cénat et al., 2021; Salari et al., 2020). These numbers indicate that the COVID‐19 pandemic has severely impacted mental health in the general population (Luo et al., 2020; Salari et al., 2020; Vindegaard & Benros, 2020).

To evaluate the impact of the pandemic on mental health, we can refer to findings from both cross‐sectional and longitudinal studies. Numerous cross‐sectional studies have suggested considerable detrimental effects of the pandemic on mental health (for a review, see, e.g., Panda et al., 2021). By contrast, longitudinal studies published thereafter have revealed mostly small and strongly heterogeneous effects on mental health symptoms after the COVID‐19 outbreak (for reviews, see e.g., Prati & Mancini, 2021; Robinson et al., 2022). Synthesising 25 longitudinal studies involving 72,004 participants from several continents, Prati and Mancini (2021) found that lockdowns overall had small effects on mental health with an overall pre‐post effect size of *g* = 0.17. Thus, most individuals appear to be psychologically resilient.

One shortcoming of these studies is that their focus on mean changes assumes similar effects of the pandemic on all people. However, from what is known about individual responses to stressful events, people tend to respond in a variety of ways to different types of disasters, such as natural disasters (e.g., hurricanes; Kessler et al., 2006) or terrorist attacks (e.g., 9/11; Farfel et al., 2008). Thus, heterogeneity in people's reactions to the pandemic should be expected (Etilé et al., 2021; Goldmann & Galea, 2014; Mancini, 2020). Most people experiencing a potentially traumatic event will not develop a significant functional impairment (but some do). A large‐scale review found that about 66% of people remained resilient when faced with adversity (Galatzer‐Levy et al., 2018). Another 21% responded with elevated symptoms but recovered quickly thereafter. Only 11% experienced chronic mental health problems after a potentially traumatic event, and 9% reported worsening mental health later on (i.e., a delayed‐onset trajectory). To be sure, these numbers suggest that a sizeable share of people—about one in five—develop mental health problems following the experience of a potentially traumatic event—but four out of five people do not. Cautiously applying these findings to the current pandemic, they suggest that we should not assume uniform effects on mental health but should instead expect effects to vary.

The vast majority of studies investigating the effects of the COVID‐19 pandemic on mental health have focussed on mean effects without addressing the possibility of heterogeneity in mental health trajectories. However, at least a small number of longitudinal studies have modelled heterogeneity in people's responses to the COVID‐19 pandemic. Data from different countries (Ireland: Hyland et al., 2021; Israel: Kimhi et al., 2021; UK: Saunders et al., 2021) suggest three to five subpopulations. Across studies, the most prevalent class was a ‘resilient’ class, but all studies also identified groups representing people with chronic or worsening mental health issues. Whereas the proportion of these groups experiencing mental health issues varied, it was sizeable in all studies (11%–32%).

Given that people differ in their reactions to the pandemic, the question that arises is: What predisposes people to develop different enduring trajectories? Previous research on natural and manmade disasters has identified several sociodemographic and personal characteristics as predictors of chronic or worsening trajectories. For example, Goldman and Galea (2014) found that being a woman, being young, having a low socioeconomic status, experiencing job loss, or previously or currently undergoing treatment for a mental health issue were associated with a higher probability of being assigned to a less favourable trajectory. By contrast, Etilé et al. (2021) found no evidence of age effects. Studies that assumed heterogeneity in mental health trajectories during the pandemic confirmed the relevance of sociodemographic variables that were previously identified as relevant for other disasters (e.g., Hyland et al., 2021; Kimhi et al., 2021). In addition, personality factors, such as neuroticism and extraversion, have been found to be associated with less favourable mental health trajectories during the pandemic (Saunders et al., 2021).

One of the most tangible consequences of the pandemic pertains to the restrictions on people's social lives. The maintenance of affiliative behaviour is known to be a protective factor when dealing with stress (Taylor, 2006), presumably also during the COVID‐19 pandemic (Mancini, 2020). However, people's opportunities to socialise have been significantly compromised by repeated and widespread measures to reduce infection rates. Therefore, increased loneliness and fewer opportunities for social support could be expected to contribute to impairments in well‐being. Not surprisingly, in studies looking at mean levels of mental health, higher levels of social support were associated with less depression and anxiety in the general population (Yin et al., 2021), less COVID‐19 anxiety in lonely people (Xu et al., 2020), and lower levels of depression and insomnia in people undergoing social isolation (Grey et al., 2020). In a review of mostly cross‐sectional studies carried out at the onset of the pandemic, Buecker and Horstmann (2021) found that, on average, mental health issues were consistently positively associated with perceived social isolation. Referring to studies that took heterogeneity in trajectories into account, we found one study that examined loneliness as a predictor of distinct trajectories (Hyland et al., 2021). In that study, loneliness predicted unfavourable mental health development from April to December 2020.

Another social factor that may be important for people's mental health in the pandemic is social comparisons. The concept of social comparisons reflects the processes by which individuals compare themselves with others with respect to behaviour and experiences (Buunk & Dijkstra, 2017; Sirgy, 2021). Downward and upward comparisons play an important role in subjective well‐being (Sirgy, 2021). Negative self‐evaluations in relation to others are associated with more symptoms of depression (Gilbert, 2000) and anxiety (McCarthy & Morina, 2020). Because individuals are more likely to employ social comparisons in threatening or uncertain situations (e.g., Kulik & Mahler, 2000), comparative judgements may have a particularly strong impact during the pandemic (Rose & Edmonds, 2021). Nevertheless, despite the observation that social media use surged during the pandemic, research on effects of social comparisons in the context of social distancing and isolation during the pandemic is scarce. In one study, downward social comparisons were associated with improvements in levels of mental health indicators (Ruggieri et al., 2021). By contrast, Yue et al. (2022) reported that both upward and downward contrasts as well as downward identification were related to higher levels of stress. Both studies looked at mean levels of mental health but did not investigate heterogeneity in mental health development across time.

We conducted a longitudinal study to examine changes in mental health. Specifically, we assessed depressive symptoms, anxiety, and stress, but also life satisfaction, as life satisfaction belongs to the positive dimension of mental health (Keyes, 2005). The study began shortly after the outbreak of the pandemic in March 2020 and lasted for 1.5 years until September 2021. Building upon previous research on different types of disasters (Farfel et al., 2008; Kessler et al., 2006), we assumed that loneliness and low social support would be associated with more pronounced detrimental changes in mental health on average (i.e., a greater increase in symptoms of anxiety, depression, and stress, and a greater decrease in life satisfaction). In addition, we believed it would be plausible to identify five latent groups representing heterogeneous mental health trajectories (Galatzer‐Levy et al., 2018). We again speculated that loneliness and social support would be associated with less favourable trajectories. Less favourable trajectories refer to overall higher levels of symptoms/lower levels of life satisfaction or increasing levels of symptoms/decreasing levels of life satisfaction (i.e., trajectories sometimes termed ‘chronic’ or ‘delayed onset’). However, given the unique situation of the pandemic, firm predictions were not possible. Hence, we regarded our analyses as exploratory.

In the following, we report on three key issues: First, we examined mean changes in mental health and life satisfaction in the total sample and across the entire 1.5 years. Second, we elucidated the heterogeneity in mental health trajectories across the first 10 months of the pandemic that was otherwise concealed by mean changes for the entire sample. Third, we investigated the role of social variables (loneliness, social support, and social comparisons) in explaining differences in both people's initial reactions to the pandemic and mental health trajectories over time.

## 2 METHOD

### 2.1 Transparency and ethical statement

The analyses of latent trajectories predicted by social factors were preregistered. The preregistration, data, *R* and *Mplus* scripts, and all materials are available at https://osf.io/jehf7/. According to German regulations, the present study was considered exempt from IRB review by the local ethics board. All procedures were in accordance with the 1964 Helsinki Declaration and its later amendments. Informed consent was obtained from all participants.

### 2.2 Participants and procedure

We conducted a longitudinal study that ranged from March 2020 to September 2021 in a large community sample. Our initial sample consisted of 2295 people who were recruited via newspaper and social media advertisements. Participation was voluntary, and we included all individuals who were 18 years of age or older. Participants were entered into a raffle for 10 vouchers worth 50€ each if they completed at least four surveys.

Participants took part in a baseline survey in March 2020. This baseline survey included sociodemographic variables, trait measures of the predictors and the outcomes, and additional items that referred to the pandemic. In the 1.5 years that followed, participants completed up to 17 additional surveys that were distributed via the SoSci Survey platform (Leiner, 2022). Here, we drew on seven of them: We used more extensive trait measurements from March 2020, March 2021, and September 2021 to inform the mean change analyses across the first 12 and the following 6 months of the pandemic. Four shorter, equidistant measurement occasions approximately spanning the first year of the pandemic (April 2020, July 2020, October 2020, January 2021) were chosen to represent each season. These four measurement occasions utilised a weekly time frame and were used to inform the latent class growth analyses. The overall compliance rate was 60.41% across the 18 months.

Participants were excluded from the sample if they did not complete the trait measures in the baseline survey (*N* = 68) or were 17 years of age or younger (*N* = 10). We further used page‐level response times and the Mahalanobis Minimum Covariance Determinant (MMCD; Leys et al., 2018) as post hoc screening criteria to detect careless or inattentive responses. In an uncontrolled environment such as online studies, inattentive responding is a considerable threat to the conclusions one can draw from study results, for example, due to attenuated correlations or additional factors that arise from careless response patterns (Meade & Craig, 2012). We computed indices for each measurement occasion separately to account for changes in sample size and participants' familiarisation with the study content. We used MMCD as a replacement for Mahalanobis distance due to MMCD's robust performance in larger samples (Leys et al., 2018). For these analyses, items with an interquartile range of zero were temporarily excluded. We removed measurement points when a participant's MMCD exceeded the critical *χ* ^2^ value (*α* = 0.001) and the respective participants completed the respective survey at least twice as fast as the median participant (Leiner, 2019). This led to the exclusion of between 0.15% and 0.56% of the measurement points (33 in total).

After excluding data, the final sample consisted of 10,128 observations from 2203 participants, resulting in an average of 4.60 out of 7 responses per participant. All models were computed using the Full Information Maximum Likelihood estimator to account for missing data. Hence, all available information has been retained.

### 2.3 Measures

#### 2.3.1 Predictor variables

##### Loneliness, emotional support, instrumental support

We used the NIH Toolbox Adult Social Relationship Scales (Cyranowski et al., 2013) to assess loneliness (2 items; e.g., ‘I feel alone and apart from others’), emotional support (four items; e.g., ‘I have someone who will listen to me when I need to talk’), and instrumental support (3 items; e.g., ‘There is someone around to help me if I need it’). For each item, response alternatives were given on a scale from 1 (never) to 5 (always). The Spearman‐Brown coefficient for two items (Eisinga et al., 2013) ranged between 0.85 and 0.87 for loneliness; McDonald's Omega ranged between 0.93 and 0.96 for emotional support, and between 0.89 and 0.91 for instrumental support.

##### Social comparison orientation

We measured participants' tendency to engage in social comparisons with six items of the Iowa‐Netherlands Comparison Orientation Measure (INCOM; Gibbons & Buunk, 1999; Schneider & Schupp, 2011). One example item reads: (‘I often try to find out what others think who face similar problems as I face.’ The response alternatives were given on a scale from 1 (strongly disagree) to 5 (strongly agree). McDonald's Omega was 0.90.

##### Better than average/worse than average

In October 2020, we created four items to measure whether participants thought they had been worse or better off than others during the pandemic. On a semantic differential (from −3 to 3), participants assessed whether their personal situation had significantly deteriorated or improved in the previous 6 months (i.e., since the pandemic outbreak), and whether they expected the situation to significantly deteriorate or improve over the coming 6 months. We then asked participants to indicate whether they believed that the situation had significantly deteriorated or improved for others and how it would develop in the future. For all analyses, we calculated a variable that is the difference between one's own situation (summarised over the previous and the coming 6 months) and other people's situation (summarised over the previous and the coming 6 months). High values on this variable represent a ‘worse‐than‐average’ effect (i.e., others are doing better than oneself during the pandemic), whereas low values represent a ‘better‐than‐average’ effect (i.e., oneself is doing better than others during the pandemic).

#### 2.3.2 Outcome variables

In our study, we used two types of questionnaires to assess the outcomes. Three more extensive measurement batteries (‘trait assessments’ in March 2020, March 2021, and September 2021) informed the mean change analyses across the first 12 and the subsequent 6 months of the pandemic. Here, we used all items from the respective scales. For the other four measurement occasions, we ad hoc shortened the questionnaires to achieve high response rates and reduce participant burden. These shorter scales, with three life satisfaction items and a total of 12 depression, anxiety, and stress items, were used to estimate the trajectories over time.

##### Life Satisfaction

Life satisfaction was measured with the German version of the Life Satisfaction Scale (Diener & Emmons, 1985; Janke & Glöckner‐Rist, 2014). One example item reads: ‘On the whole, my life this past week has been what I want it to be.’ For each item, response alternatives were given on a 7‐point Likert scale from 1 (strongly disagree) to 7 (totally agree). McDonald's Omega ranged between 0.90 and 0.94.

##### Depression, anxiety and stress

We used items derived from the Depression, Anxiety and Stress Scale (DASS‐21; Nilges & Essau, 2015) to measure depressive symptoms (e.g., ‘I felt sad and depressed’), anxiety (e.g., ‘I felt scared without any good reason’), and stress (e.g., ‘I noticed that I was getting agitated pretty quickly’). For each item, response alternatives were given on a 4‐point Likert scale from 1 (did not apply to me at all) to 4 (applied to me very strongly, or most of the time). McDonald's Omega ranged between 0.76 and 0.82 for depression, between 0.75 and 0.79 for anxiety, and between 0.77 and 0.81 for stress.

The complete item wordings and a description of the coding of the variables is given in the code book on the OSF. We present the means, standard deviations, reliabilities, and zero‐order correlations for the baseline survey in Table 1.

**TABLE 1:** Means, standard deviations, and zero‐order correlations for all study variables

		*M* (*SD*)	Omega	2.	3.	4.	5.	6.	7.	8.	9.
1.	Loneliness	2.23 (0.96)	0.86	−0.48	−0.40	0.23	0.03	0.60	0.38	0.36	−0.51
2.	Emotional support	4.22 (0.84)	0.83	–	0.53	0.00	−0.04	−0.42	−0.28	−0.17	0.44
3.	Instrumental support	4.15 (1.02)	0.88		–	−0.03	−0.06	−0.32	−0.18	−0.10	0.39
4.	Comparisons	3.08 (0.88)	0.90			–	−0.05	0.24	0.24	0.31	−0.10
5.	Worse than average	−1.06 (2.07)	–				–	0.06	0.04	−0.01	−0.06
6.	Depressive symptoms	1.79 (0.63)	0.76 ‐ 0.82					–	0.63	0.56	−0.65
7.	Anxiety	1.71 (0.57)	0.75 ‐ 0.79						–	0.54	−0.40
8.	Stress	2.29 (0.64)	0.77 ‐ 0.81							–	−0.33
9.	Life satisfaction	5.09 (1.16)	0.90 ‐ 0.94								​–

*Note*: *N* = 2203. We report Spearman Brown coefficients for the reliability of loneliness because it consists of only two items. All reported correlation coefficients are Pearson correlations. Correlation coefficients equal to or larger than |0.06| were significant at an *α* level of 0.05.

### 2.4 Statistical procedure

All statistical analyses were carried out using either R 4.1.2 (R Core Team, 2021) or Mplus Version 8.7 (Muthén & Muthén, 2018) via MplusAutomation (Hallquist & Wiley, 2021).

#### 2.4.1 Latent change models

To examine initial responses to the pandemic, intraindividual changes across the first 12 months of the pandemic (March 2020 to March 2021; ‘Phase 1’ in the following), and intraindividual changes across the next 6 months of the pandemic (March 2021 to September 2021; ‘Phase 2’ in the following), we applied the latent change model (LCM; Steyer et al., 1997) approach. In LCMs, changes in scores are represented as latent factors, thus taking measurement error into account. These latent factors can be correlated with predictors and other change variables, thus allowing for a flexible modelling approach. A requirement of the models is that the assumption of strong measurement invariance over time holds in the data (Meredith, 1993).

**FIGURE 1:**
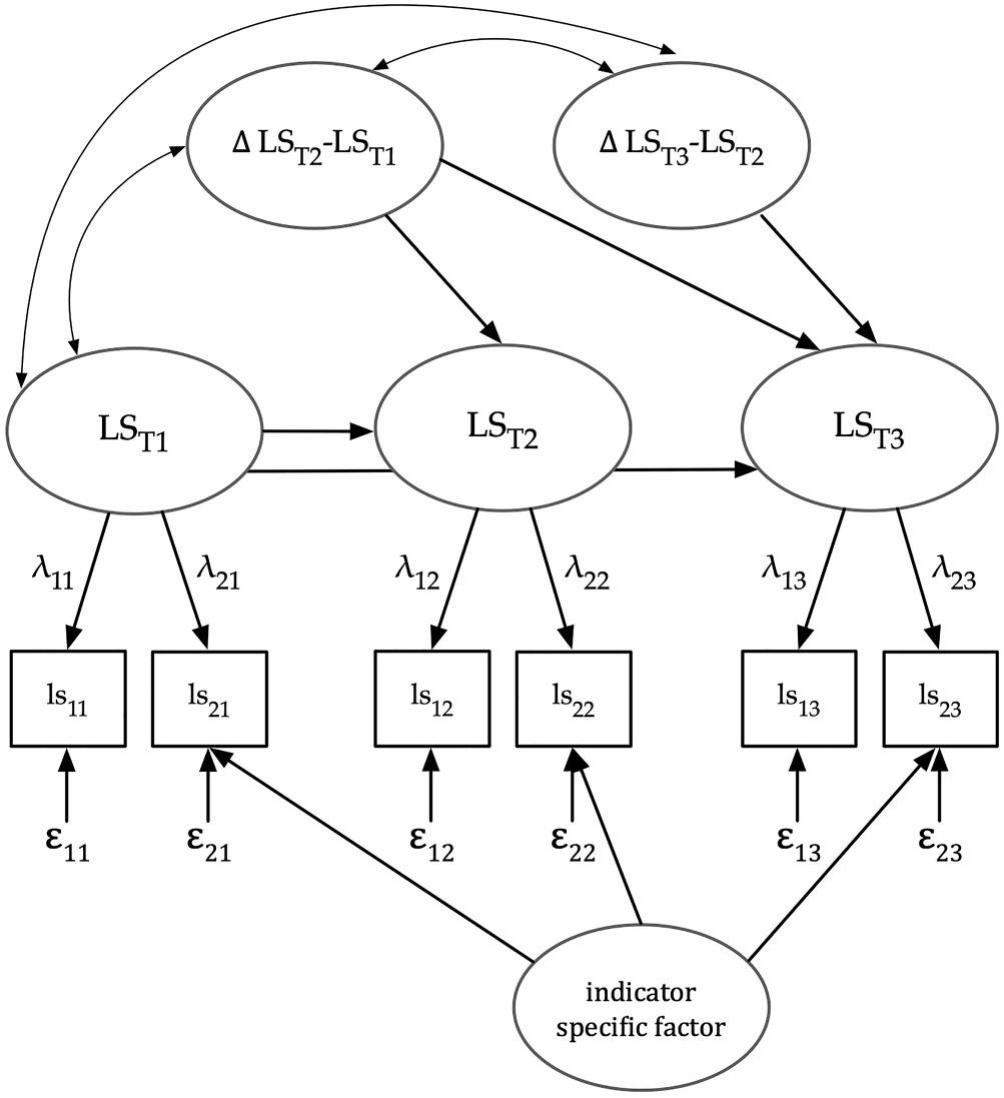
Schematic neighbor latent change model for life satisfaction. Indicators ls_11_ to ls_23_ denote item parcels (two item parcels per measurement occasion). The measurement model operates under strong measurement invariance (loadings and intercepts of the first and second indicators at each measurement occasion were constrained to be equal over time). To account for uniqueness in the second indicator shared over time, we included an indicator‐specific factor. In the neighbor change variant of the LCM, latent change scores represent interindividual differences in the intraindividual change from measurement occasion t‐1 to measurement occasion t. This figure depicts the change model for life satisfaction, but the same setup was used for all four outcomes in our study

With our data, we applied the neighbor change version of the latent change model with change score variables reflecting true score changes between adjacent measurement occasions in all outcomes of interest (i.e., parallel changes). First, we computed an unconditional LCM including change scores for all variables and their respective correlations but no predictors. Second, we computed a conditional LCM using social variables as predictors. Hence, in the conditional LCM, we looked at potential risk and protective factors over and above the effects of the other variables in the respective block. The models were evaluated via multiple fit indices (Hu & Bentler, 1999) with the comparative fit index (CFI) ≥ 0.95, and the root mean square error of approximation (RMSEA) ≤ 0.08 reflecting acceptable model fit. To account for missing data typical in longitudinal studies, we used full information maximum likelihood (FIML) estimation. We show the full representation of the measurement model in Figure 1. For further details about the latent change analyses, see the Online Materials on the OSF.

#### 2.4.2 Latent class growth analyses

To examine variability in mean level change across the first 10 months of the pandemic, we applied Latent Class Growth Analyses (LCGAs, Muthén & Muthén, 2000). LCGAs are designed to explain heterogeneity in change over time by modelling latent classes of individuals with distinct trajectories. By allowing for post hoc class identification, LCGAs use an iterative estimation process to uncover underlying subpopulations that were not known a priori. With the estimated model parameters, researchers can derive an individual person's probability of belonging to a specific class. Given that the estimates are probable representations of the underlying groups, LCGAs are inherently exploratory.

In LCGA, each class is represented by its own baseline levels (i.e., intercept) and rates of change (i.e., slope), to which individuals in the sample are probabilistically allocated. Since all members of one class are assumed to follow the same trajectory, the variances of the latent factors (i.e., intercept and slope) in each class are constrained to zero. We successively increased the number of classes. Based on theoretical assumptions we pre‐registered five distinct classes for each outcome^1^ and hence aimed to estimate up to six class solutions. Empirically, the number of classes can be increased until the model encounters convergence issues. To establish global maxima and avoid class solutions based on local maxima, we implemented 200 sets of random start values and the best 50 of these starts were kept for final stage optimisation. By comparing specifications with different numbers of classes, the relative fit of the class solutions was determined. We compared the relative fit of the class solutions using Akaike Information Criterion (AIC), Bayesian Information Criterion (BIC), and Sample‐Size Adjusted Bayesian Information Criterion (ssBIC or aBIC). Because the information criteria tend to decrease for each additional class up to choosing a saturated model, we followed the recommendations of Petras and Masyn (2010) and visually displayed the values of the information criteria to check for an ‘elbow’ indicating a point of diminishing returns in model fit improvements (figures are provided in the Online Materials on the OSF).^2^ In sum, the final class solution for each outcome was chosen based on statistical criteria (information criteria and likelihood‐based tests including a visual inspection), and our theoretically grounded evaluation of the estimations results in terms of interpretability (Bauer & Curran, 2003).

**FIGURE 2:**
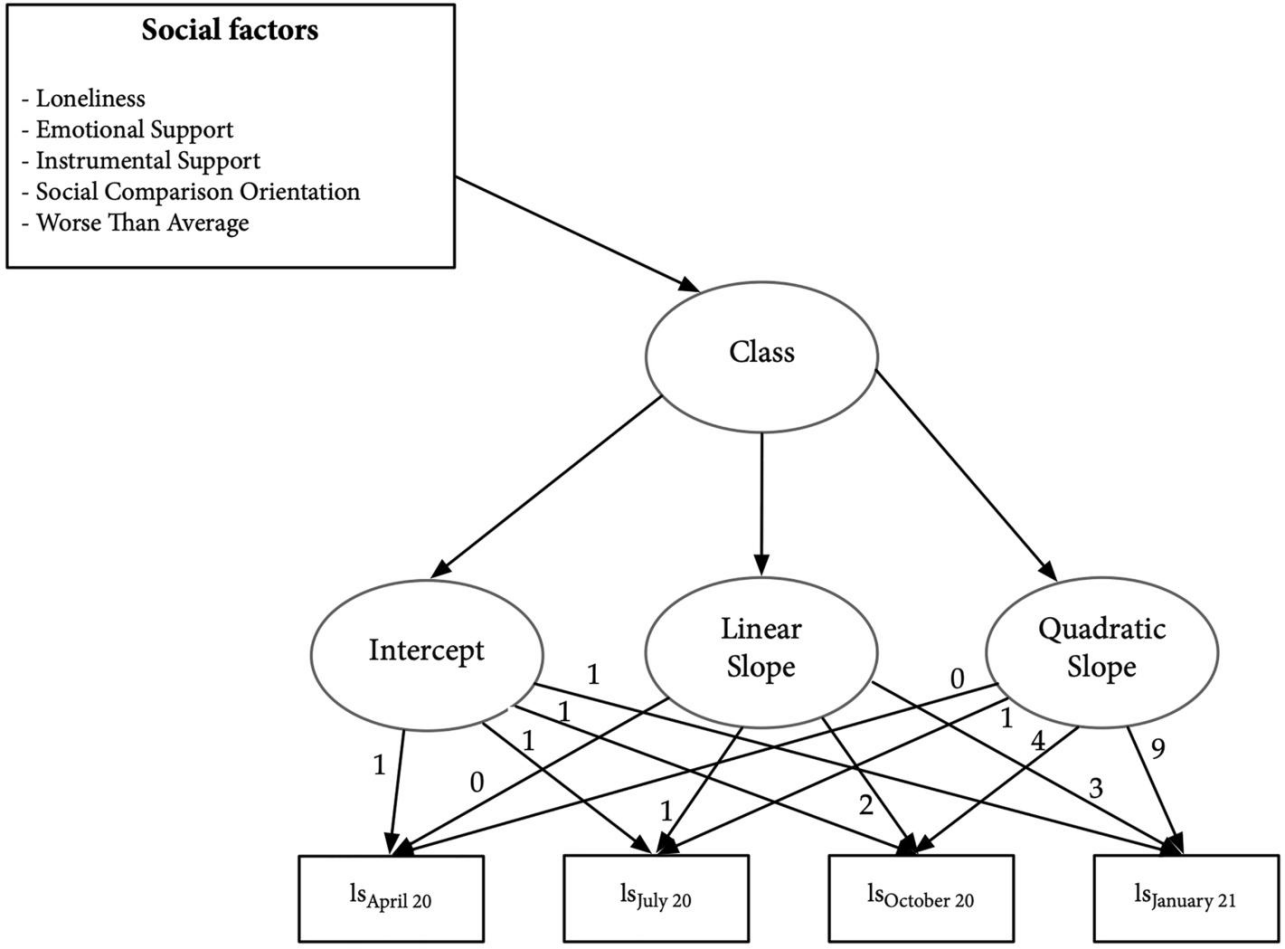
Schematic conditional latent class growth model with the predictors

To test the associations between social factors and the classes represented by the different trajectories, we used the three‐step method to ensure that our predictors and covariates did not influence the relative class sizes (Vermunt, 2010). More specifically, this three‐step approach takes into account that the class allocations are not the true class memberships but contain classification errors instead (Van de Schoot et al., 2017). In these analyses, Mplus reduces the sample to participants with complete covariates. We show a schematic representation of the LCGA model with predictors in Figure 2. For additional details on the LCGA procedures see Online Materials on the OSF.

## 3 RESULTS

Participants in this study were 78.2% female (21.8% male), with an average age of 38.63 years (*SD* = 14.09, Range 18–90 years); 71.74% of participants were in a relationship; and 31.73% had at least one child (*M* = 1.58 children). About one in five participants belonged to the COVID‐19 risk group (21.33%, *N* = 470), and 29.01% (*N* = 639) reported having a previous or current mental health diagnosis. To assess socioeconomic status, we asked participants to place themselves on a 10‐rung ladder, with the highest rung representing the people with the most money, the most education, and the most respected jobs (Adler et al., 2000). On average, participants reported a socioeconomic status of 6.14 (*SD* = 1.58, Range 1–10).

### 3.1 Initial responses and intraindividual changes in mental health and life satisfaction

#### 3.1.1 Mean initial responses and changes

The unconditional LCM with strong measurement invariance over time showed an excellent fit to the data, *χ* ^2^(156, *N* = 2203) = 384.35, *p* < 0.001, CFI = 0.99, RMSEA = 0.026. In this model, we found that on average, depressive symptoms increased slightly in Phase 1 (*e* = 0.061, *p* < 0.001, *d* = 0.13) and did not change further in Phase 2 (*e* = −0.003, *p* = 0.850, *d* = −0.01). Average stress levels did not change reliably in either phase (Phase 1: *e* = −0.003, *p* = 0.858, *d* = −0.01; Phase 2: *e* = 0.020, *p* = 0.121, *d* = 0.06). By contrast, anxiety decreased in Phase 1 (*e* = −0.043, *p* = 0.002, *d* = −0.11) and remained at this level in Phase 2 (*e* = 0.006, *p* = 0.621, *d* = 0.02). Finally, life satisfaction decreased substantially in Phase 1 (*e* = −0.276, *p* < 0.001, *d* = −0.31), followed by a substantial increase in Phase 2 (*e* = 0.177, *p* < 0.001, *d* = 0.25).

All change scores varied significantly (all *p*s < 0.001), indicating that the outcome variables changed in different ways in different people. Temporally parallel changes (i.e., changes in different outcome variables occurring during the same time frame) were substantially correlated in Phase 1 (all *r*s |0.32| to |0.68|) and in Phase 2 (all *r*s |0.26| to |0.70|).

#### 3.1.2 Predictors of initial responses and changes

In a second LCM, we included the social factors of loneliness, emotional support, instrumental support, social comparisons, and better‐than‐average scores as (a) covariates of initial responses and (b) predictors of change. This model showed an excellent fit to the data, *χ* ^2^(215, *N* = 2203) = 926.99, *p* < 0.001, CFI = 0.98, RMSEA = 0.04. We present the results for all predictors in Table 2.

**TABLE 2:** Predictors of model‐implied initial status and latent change scores

	D T_1_	ΔD T_2_‐T_1_	ΔD T_3_‐T_2_	A T_1_	ΔA T_2_‐T_1_	ΔA T_3_‐T_2_	S T_1_	ΔS T_2_‐T_1_	ΔS T_3_‐T_2_	LS T_1_	ΔLS T_2_‐T_1_	ΔLS T_3_‐T_2_
Loneliness	0.48*	−0.01	−0.12*	0.32*	−0.01	−0.04	0.28*	−0.00	−0.11	−0.38*	−0.03	0.14*
Emotional support	−0.22*	0.00	−0.04	−0.16*	−0.01	−0.03	−0.05	−0.06	−0.03	0.19*	0.02	0.06
Instrumental support	−0.01	0.10*	−0.11*	0.02	0.10*	−0.06	0.08*	0.03	−0.01	0.12*	−0.11*	0.08
Comparisons	0.12*	−0.02	0.03	0.20*	−0.02	−0.04	0.24*	0.02	−0.02	−0.05	0.09*	−0.04
Worse than average	0.10*	−0.09*	0.04	0.04	−0.04	−0.03	−0.03	−0.04	−0.07	−0.04	0.08*	​‐0.06

*Note*: Model‐implied prediction based on *N* = 2203. D T_1_ = Depression at T_1_. A T_1_ = Anxiety at T_1_. S T_1_ = Stress at T_1_. LS T_1_ = Life Satisfaction at T_1_. All coefficients in the table are standardised estimates.

*p < 0.05.

Looking at the initial responses, we found that higher levels of general loneliness were associated with impaired mental health and lower life satisfaction (all βs |0.28| to |0.48|). People reporting higher levels of emotional support experienced less anxiety, fewer depressive symptoms, and more life satisfaction (all βs |0.16| to |0.22|), whereas high levels of instrumental support were associated with more stress and more life satisfaction (*β* = −0.08 and *β* = 0.12 respectively). Individuals who tend to frequently compare themselves with others reported higher levels of stress, anxiety, and depression at T1 (all βs |0.12| to |0.24|), whereas perceptions of being worse off than others during the pandemic were linked to higher levels of depression only (*β* = 0.10).

Looking at the changes over time, we found that higher levels of loneliness were associated with a larger decrease in depressive symptoms and a larger increase in life satisfaction in Phase 2 (*β* = −0.12 and *β* = 0.28 respectively). Higher levels of instrumental support predicted a larger increase in depressive symptoms in Phase 1 (*β* = 0.10) and a larger decrease in depressive symptoms in Phase 2 (*β* = −0.11). In line with this, higher instrumental support was also associated with a larger decrease in life satisfaction (*β* = −0.11) and a smaller decrease in anxiety in Phase 1 (*β* = 0.10). By contrast, we found no evidence that emotional support would predict changes in mental health and life satisfaction in either Phase 1 or 2 (all βs < |0.06|). The tendency to compare oneself with others and a feeling of being worse off than others were associated with smaller decreases in life satisfaction in Phase 1 (*β* = 0.09). In addition, feeling worse off than others was linked to a smaller increase in depression in Phase 1 (*β* = −0.09).

### 3.2 Trajectories of mental health and life satisfaction

**FIGURE 3:**
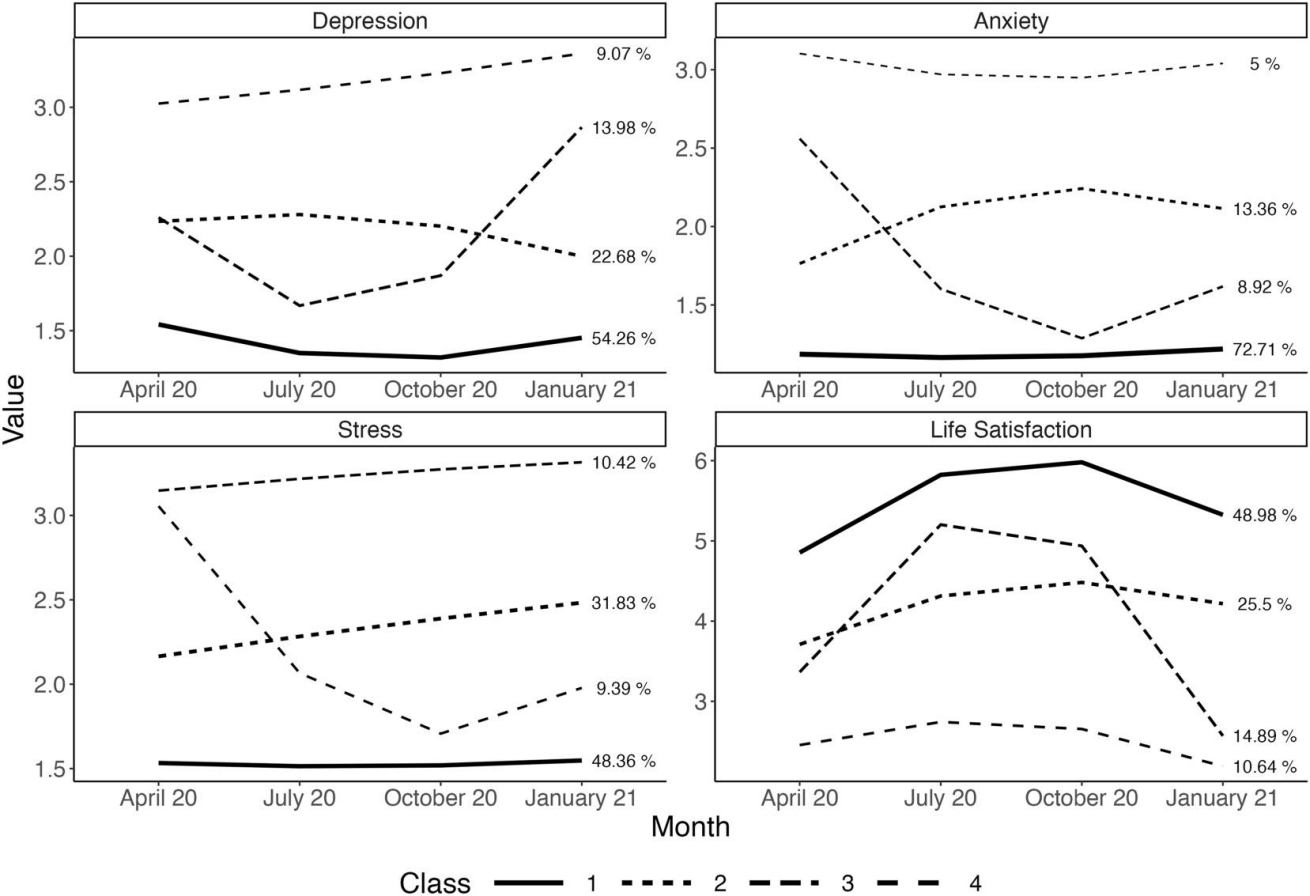
Latent class growth trajectories of depressive symptoms, anxiety, stress, and life satisfaction. Class 1 represents the largest class for each outcome. The figure depicts the time period ranging from April 2020 to January 2021

In the second part of the analyses, we modelled the trajectories of the three mental health outcomes and life satisfaction across time (Table 3). All outcomes followed four trajectories each (Figure 3). We then examined whether the social factors predicted participants' specific trajectory. For odds ratios for all predictors in all models, see the Online Materials on the OSF.

**TABLE 3:** Fit indices in the LCGA models for life satisfaction, depressive symptoms, anxiety and stress

	LL	AIC	BIC	aBIC	Entropy	BLRT	Class 1	Class 2	Class 3	Class 4	Class 5	Class 6
Life satisfaction												
1 Class	−9976.00	19,965.99	20,005.37	19,983.13	1.00	‐	100%					
2 Classes	−9468.02	18,958.03	19,019.91	18,984.96	0.65	−9976.00***	71%	29%				
3 Classes	−9345.99	18,721.99	18,806.37	18,758.71	0.60	−9468.02***	50%	39%	11%			
4 Classes	−9306.94	18,651.88	18,758.76	18,698.39	0.57	−9345.99***	49%	25%	15%	11%		
5 Classes	−9279.40	18,604.80	18,734.17	18,661.10	0.50	−9306.94***	34%	27%	17%	13%	9%	
6 Classes	−9268.46	18,590.92	18,742.80	18,657.02	0.51	−9279.40***	31%	29%	16%	11%	9%	5%
Depressive symptoms												
1 Class	−6264.15	12,542.30	12,581.67	12,559.43	1.00	‐	100%					
2 Classes	−5572.75	11,167.50	11,229.38	11,194.43	0.74	−6264.15***	73%	27%				
3 Classes	−5360.80	10,751.59	10,835.97	10,788.31	0.69	−5572.75***	56%	34%	9%			
4 Classes	−5287.60	10,613.20	10,720.08	10,659.72	0.66	−5360.80***	54%	23%	14%	9%		
5 Classes	−5241.55	10,529.10	10,658.48	10,585.41	0.66	−5287.60***	52%	15%	14%	10%	9%	
6 Classes	−5202.78	10,459.57	10,611.45	10,525.66	0.66	−5241.55***	48%	15%	13%	9%	9%	6%
Anxiety												
1 Class	−5484.79	10,983.57	11,022.95	11,000.71	1.00	‐	100%					
2 Classes	−4424.58	8871.15	8933.03	8898.08	0.90	−5484.79***	85%	15%				
3 Classes	−4187.47	8404.94	8489.32	8441.66	0.84	−4424.58***	75%	18%	7%			
4 Classes	−3969.41	7976.82	8083.70	8023.34	0.87	−4187.47***	73%	13%	9%	5%		
5 Classes	−3841.63	7729.26	7858.64	7785.56	0.83	−3969.41***	68%	10%	8%	8%	6%	
6 Classes	−3686.35	7426.70	7578.58	7492.80	0.89	−3841.63***	62%	18%	7%	6%	4%	3%
Stress												
1 Class	−6479.08	12,972.17	13,011.54	12,989.30	1.00	‐	100%					
2 Classes	−5797.40	11,616.80	11,678.68	11,643.73	0.69	−6479.08***	68%	32%				
3 Classes	−5636.71	11,303.43	11,387.80	11,340.15	0.65	−5797.40***	50%	38%	11%			
4 Classes	−5582.16	11,202.32	11,309.20	11,248.83	0.68	−5636.71***	48%	32%	10%	9%		
5 Classes	−5530.13	11,106.27	11,235.65	11,162.57	0.62	−5582.16***	33%	33%	15%	12%	7%	
6 Classes	−5491.64	11,037.27	11,189.15	11,103.37	0.61	−5530.13***	32%	30%	14%	9%	8%	7%

*Note*: *N* = 2063.

Abbreviations: aBIC, sample‐size adjusted BIC; AIC, Akaike information criterion; BIC, Bayesian information criterion; BLRT, Bootstrap likelihood ratio test; LCGA, latent class growth analysis; LL, Model log likelihood.

***p < 0.001.

#### 3.2.1 Depressive symptoms

The largest class showed a continuously low trajectory (Class 1, 54%). In this class, the likelihood of experiencing depressive symptoms remained permanently close to zero. The second trajectory (Class 2, 23%) showed a mostly linear trend at slightly elevated levels of depressive symptoms. In this class, depression levels moderately decreased from summer to winter. The third, U‐shaped trajectory (Class 3, 14%) began with modest levels of depressive symptoms, improved during the summer, and showed a large increase in symptoms afterwards. Finally, the fourth trajectory (Class 4, 9%) showed the overall highest levels of depressive symptoms and a steady increase throughout the study period.

Using the largest class as a reference group, higher levels of loneliness were associated with the trajectories representing Classes 2 and 4, whereas higher levels of emotional support were associated with the trajectory representing Class 4. The tendency to compare oneself with others was associated with the trajectory representing Class 3.

#### 3.2.2 Anxiety

Class 1 (73%) had a continuously low likelihood of anxiety symptoms close to zero. The second trajectory (Class 2, 13%) showed a steady increase from the beginning of the pandemic until October 2020 with anxiety levels remaining elevated thereafter. In the third class (9%), the initial levels of anxiety dropped rapidly from April to October 2020 and increased slightly again in winter. The fourth trajectory (Class 4, 5%) experienced constantly high levels of anxiety with negligible changes during the study period.

Using the largest class as a reference group, higher levels of loneliness were associated with the trajectories representing Classes 2 and 4. Emotional support was associated with the trajectory representing Class 2, whereas the tendency to compare oneself with others was associated with the trajectories representing Classes 2, 3, and 4.

#### 3.2.3 Stress

Class 1 (48%) had a permanently low likelihood of stress symptoms with almost no changes during the study period. The second trajectory (Class 2, 32%) showed a small but steady increase from the beginning of the pandemic until January 2021. Class 3 (10%) had a continuously high trajectory with a small increase throughout the study period. The fourth trajectory (Class 4, 9%) began with high levels of stress symptoms, which decreased a great deal until fall, and reported a small increase from fall to winter.

Using the largest class as a reference group, higher levels of loneliness and a stronger tendency to compare oneself with others were associated with the trajectories representing Classes 2, 3, and 4. Higher levels of emotional support were associated with the trajectory representing Class 3.

#### 3.2.4 Life satisfaction

The first trajectory (Class 1, 49%) covaried with the course of the pandemic: Life satisfaction was lower at the onset of the pandemic in April 2020, improved over the summer, and decreased again in January 2021. Participants in this class had the highest overall levels of life satisfaction. The second class (25%) reported the lowest life satisfaction at the beginning of the pandemic and subsequently showed a linear increase until fall 2020 with a slight decrease in January 2021. The third trajectory (Class 3, 15%) showed an inverted U‐shape similar to Class 1 but more strongly pronounced and with more modest levels of life satisfaction. Unlike Class 1, participants in Class 3 experienced a stark decrease in life satisfaction in January 2021. Class 4 (11%) exhibited a continuously low trajectory with slight improvements during summer and a further reduction in life satisfaction in winter 2021.

Using the largest class (Class 1) as a reference group, higher levels of loneliness were associated with the trajectories representing Classes 2 and 4. Higher levels of emotional support were associated with the trajectories representing Classes 3 and 4, whereas high instrumental support was associated with the trajectory representing Class 3. Finally, feeling worse off than other people was associated with the trajectory representing Class 3.

## 4 DISCUSSION

The current large‐scale longitudinal study was aimed at deepening our understanding of how the COVID‐19 pandemic affected mental health and life satisfaction and how these outcomes changed across the 1.5 years following the outbreak of the pandemic. The longitudinal design, along with sophisticated statistical analyses, allowed us to identify four distinct developmental trajectories of mental health and life satisfaction across the study period. In addition, the study provides a unique focus on how social factors predict membership in one of the four classes represented by these four developmental trajectories.

### 4.1 (Heterogeneous) changes in mental health

In the first year of the pandemic, depression scores increased slightly on average and then maintained their level. At the same time, about 50% of the sample was assigned to a group that showed no symptoms of depression. However, almost 10% of the sample reported high—and slightly increasing—levels of depressive symptoms, suggesting that the pandemic had the strongest effects on people who were already vulnerable to depression. Another 14% of the sample reported moderate levels of depressive symptoms at first but showed a large increase in symptoms from October 2020 to January 2021. This development corresponds to the development of the pandemic in Germany at that time: Infection rates were rising sharply, and political measures were taken to flatten the curve by placing restrictions on public life.

On average, anxiety decreased in the first year and did not change thereafter. Broken down by group, about three out of four people in our sample hardly felt any symptoms of anxiety. At the same time, the first measurement point already represented an acute reaction to the pandemic, which may explain why anxiety decreased slightly over time. People likely reacted with symptoms of anxiety to the onset of the pandemic and the uncertainty of a potentially threatening situation. Gradually, people's perceptions of the pandemic as something threatening may have diminished, and thus, so may have anxiety symptoms.

Stress levels did not change on average during the first 18 months of the pandemic. In line with this finding, about half of our participants reported consistently low stress. One third of participants reported a small but steady increase in stress in the first year of the pandemic, consistent with the mean‐level results on anxiety. Interestingly, almost 10% of our sample reported large decreases in stress levels from April to October 2020—a trend clearly covarying with the overall situation in Germany in terms of incidence rates and restrictions.

Life satisfaction showed the greatest mean changes by decreasing in the first year but then increasing again. Indeed, the life satisfaction of half of the participants covaried with the course of the pandemic with lower levels in April 2020 and January 2021 and higher levels in July and October 2020. For about 15% of the sample, we saw a worrying trend from October 2020 to January 2021 with a stark decrease in life satisfaction. This development suggests that the perspective of being in a pandemic in winter may have worsened people's evaluations of their lives.

Overall, in line with previous research (e.g., Saunders et al., 2021; Solbakken et al., 2021), our results indicate that objective improvements in the pandemic situation, such as the availability of vaccines and (at least temporary) returns to normal daily life, might not have led to the desired rapid improvement in mental health in the population—at least not for everyone. This finding is worrying, given that the changes reported in this study used the initial reaction to the pandemic (i.e., already increased levels of mental health symptoms and reduced life satisfaction) as a reference point. This means that a considerable proportion of our participants did not return to the levels of overall well‐being reported in March 2020. Therefore, they certainly did not return to the even higher levels of well‐being that likely existed before the pandemic. At the same time, many people were not strongly affected by the pandemic—a finding that is often reflected in the small effect sizes from studies that look only at mean changes. However, those who were affected and at risk were more likely to experience even more severe consequences.

### 4.2 Social factors as predictors of changes in mental health

For practitioners and in view of potential future lockdowns, it is particularly important to identify vulnerability and risk factors. Whereas previous research identified several risk factors from the domain of sociodemographic and personality variables, we focussed on the role of social factors in the development of mental health and life satisfaction over time.

Overall, people who felt lonely at the onset of the pandemic had a higher risk of following a worse course of mental health. Emotional support acted as a protective factor. Instrumental support, by contrast, was ambiguous and mostly maladaptive: On average, higher levels of instrumental support predicted a larger increase in depression, a greater decrease in life satisfaction, and a greater increase in anxiety. Whereas instrumental support served as a buffer against stress in pre‐pandemic times (Thoits, 2011; but see Gur‐Yaish et al., 2013), emotional and instrumental support may have opposing effects on mental health during the pandemic. Consistent with this idea, social support was negatively associated with worry, whereas instrumental support had an inverse relationship with general worry in a previous study (Zysberg & Zisberg, 2020).

The tendency to compare oneself with others turned out to be a consistent risk factor. The subjective assessment of whether one was worse off than others during the pandemic was generally substantially correlated with depression but apart from that, had negligible influences on mental health. Together, these findings suggest that the tendency to compare one's situation with others per se puts people at risk, as opposed to the specific outcomes of these comparisons, such as feeling worse off than others.

### 4.3 Limitations and future research

We need to qualify our conclusions in light of several limitations. First, although the study was picked up by transregional newspapers across Germany and achieved a decent sample size, the sample remains nonrepresentative. Therefore, we cannot rule out the possibility that our sampling procedure introduced a bias in participants who were either very affected or not at all affected by the pandemic. Also, our advertisement may have attracted more women, resulting in a predominantly female sample, in line with most online studies (Smith, 2008). However, the large proportion of women might have affected the relative class sizes reported in our study. Being female has been an important vulnerability factor during the COVID‐19 pandemic (e.g., Saunders et al., 2021; see also Online Materials, p. 2), implying that class solutions representing detrimental mental health trajectories might have been smaller in a more male sample. Second, the data were collected only in Germany. A review of studies conducted early in 2020 found no evidence that country‐level factors, such as the number of COVID cases or government measures, could explain heterogeneity in primary studies (Robinson et al., 2022). However, our data might still not be generalisable to other countries. Third, the study lacks pre‐pandemic baseline data. Hence, our baseline must be understood as reflecting an acute reaction to the COVID‐19 pandemic with most likely increased levels of mental health symptoms and reduced life satisfaction. Consequently, we must be careful when describing and interpreting the change scores: It is possible that the pre‐pandemic average levels of mental health symptoms were lower and those of life satisfaction were higher than our baseline. Finally, our last measurement point reflects mental health and life satisfaction in fall 2021 and does not cover the (worsened) situation in Germany in winter 2021/22. Thus, our effect sizes are conservative estimates of the true changes that likely occurred across this period.

Future research could keep following diverse samples of participants to track mental health changes across longer periods of time and examine whether the emerging heterogeneity in responses to the pandemic increases or decreases further. The different roles of instrumental and emotional support during mandatory social distancing on people's well‐being should also be explored further. In our study, comparisons with others were a constant risk factor. Future studies could examine what influences the extent to which people compare themselves with other people in response to stress and how the negative effects of social comparisons can be mitigated. Practitioners could pay more attention to the aspect of loneliness in interventions. During mandatory social distancing, online interventions, in particular, could be useful for providing support in people's daily lives. At the same time, care must be taken to ensure that developing a sense of connectedness with others (e.g., through social media) does not lead to increased social comparisons.

#### 4.3.1 Conclusion

This study found that mental health symptoms and life satisfaction showed heterogeneous developmental trajectories across the first 10 months of the pandemic. Although depressive symptoms increased and life satisfaction decreased on average in the first year of the pandemic, a substantial portion of our sample was largely unaffected during this period. However, vulnerable groups exist and should be provided with targeted economic and psychosocial resources to help them bounce back. Of the social factors, loneliness at the onset of the pandemic and the propensity to engage in social comparisons were identified as risk factors. During a pandemic, people seem to benefit from emotional—but not from instrumental—social support.

## CONFLICT OF INTEREST

The authors have declared that they have no conflict of interest.

## Data Availability

The data that support the findings of this study are openly available in the OSF at https://osf.io/jehf7/

## ENDNOTES

^1^
Overall, this analytical procedure deviated from our preregistration (OSF) for several reasons. First, latent change models were not part of the preregistration. For latent classes, we encountered estimation problems (e.g., convergence issues, local maxima) with more complex models (e.g., the mean and covariance models) Hence, we decided to retain LCGAs only and also reduced the number of measurement points to four per year per construct. In addition, we planned to use the bootstrap likelihood ratio test (BLRT) to compare neighbouring class models. However, the BLRT was significant for all class solutions and was thus uninformative.

^2^
We want to thank the anonymous reviewer for suggesting LCGAs with parallel processes. In this paper, when running univariate LCGAs, we focused on estimating trajectories in the four outcomes independently from each other. However, an LCGA with parallel processes provides different but valuable insights. We present the results of this analysis in the Online Materials on the OSF.
